# Data on changes in the fatty acid composition during fruit development and ripening of three mango cultivars (Alphonso, Pairi and Kent) varying in lactone content

**DOI:** 10.1016/j.dib.2016.09.018

**Published:** 2016-09-20

**Authors:** Ashish B. Deshpande, Hemangi G. Chidley, Pranjali S. Oak, Keshav H. Pujari, Ashok P. Giri, Vidya S. Gupta

**Affiliations:** a Plant Molecular Biology Unit, Division of Biochemical Sciences, CSIR-National Chemical Laboratory, Pune 411008, India; b Dr. Balasaheb Sawant Konkan Agriculture University, Dapoli 415712, India

**Keywords:** α-linolenic acid, Fatty acids, Flavor, Lactones, Mango cultivars

## Abstract

Data in this article presents fatty acid composition of three mango cultivars; Alphonso, Pairi and Kent through fruit development and ripening. Change in the ω-6 and ω-3 fatty acids level during mango fruit development and ripening is depicted. Also, data on aroma volatile ‘lactones’ composition from pulp and skin tissues of these cultivars at their ripe stage, respectively is provided. Statistical data is also shown, which correlates modulation in lactone content with that of fatty acid composition and content during fruit development and ripening in all the three mango cultivars.

**Specifications Table**

**Table:** 

Subject area	*Biology, Chemistry*
More specific subject area	*Fatty acid and lactone composition of mango fruit*
Type of data	*Table, graph, figure*
How data was acquired	*GC–MS: 7890B GC system Agilent Technologies coupled with Agilent 5977A MSD (Agilent technologies*^®^, *CA, U.S.A.)*
Data format	*Analyzed*
Experimental factors	*Pulp and skin tissues from fruits of three cultivars collected at various fruit development and ripening stages, tissue was snap frozen and stored at* −80 °C.
Experimental features	*Fatty acid methyl esters (FAMEs) were synthesized by transesterification and aroma volatiles were extracted in dichloromethane, identified and quantified by GC–MS*
Data source location	*Plant Molecular Biology Unit, Division of Biochemical Sciences, CSIR-National Chemical Laboratory, Pune 411008, (M. S.) India.*
Data accessibility	*Data is provided within this article*

**Value of the data**

• Data from this research highlights the increase in the unsaturated fatty acid content in pulp and skin of mango with ripening and making ripened fruits more nutritious.
• Data signifies nutritionally important ω-3 fatty acid (α-linolenic acid) rich nature of mango skin for utilization by various industries.
• Investigated data from this article reveals probable fatty acid precursors for biosynthesis of lactones, which is useful to the researchers working in the area of flavor biochemistry.

## 1 Data

**Fig. 1:**
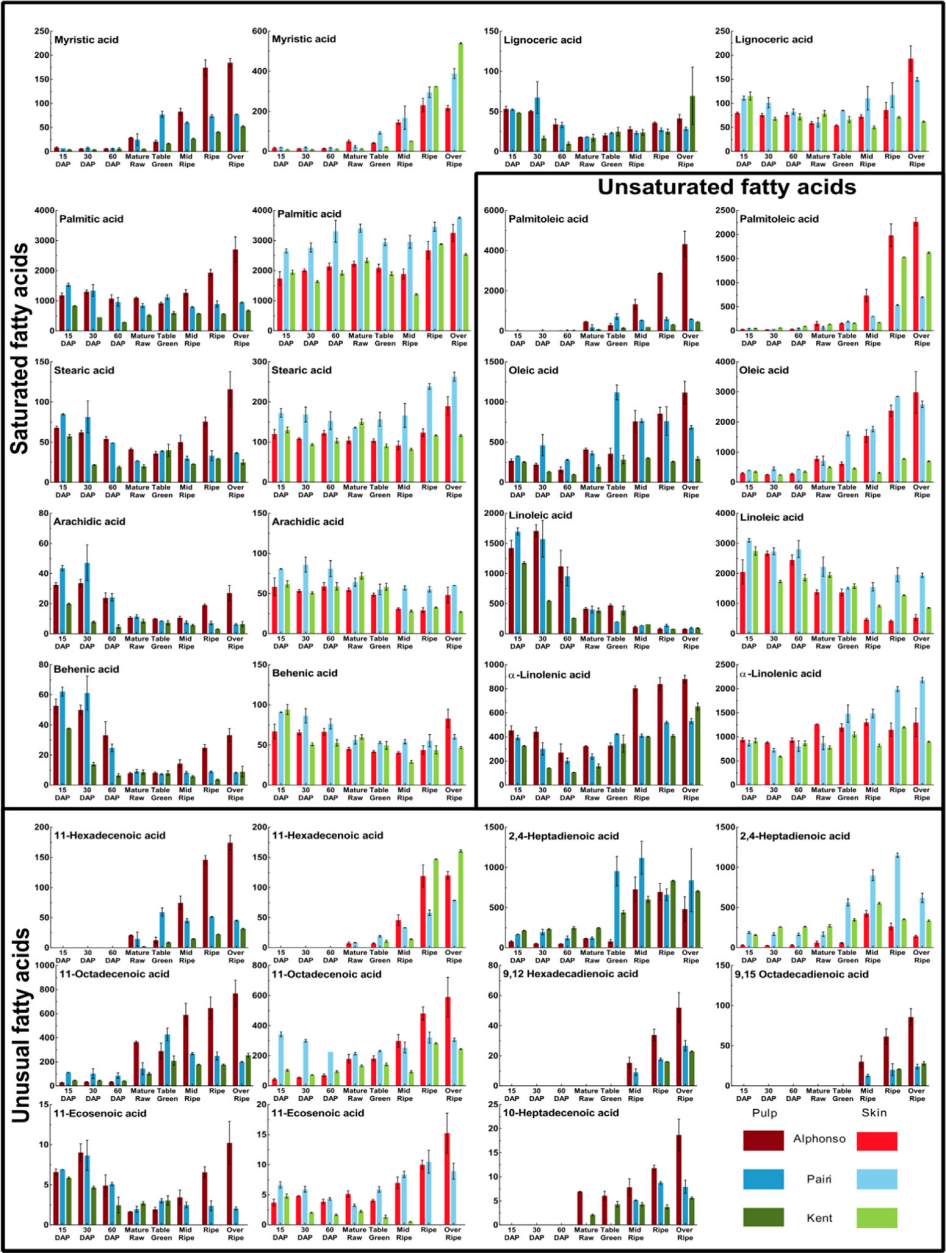
Fatty acid column chart- Content (µg g^−1^) of individual fatty acid from pulp and skin through various developing and ripening stages of Alphonso, Pairi and Kent mango cultivars. Vertical bars at each data point represent standard error of measurement calculated for the biological replicates used in the study.

**Fig. 2:**
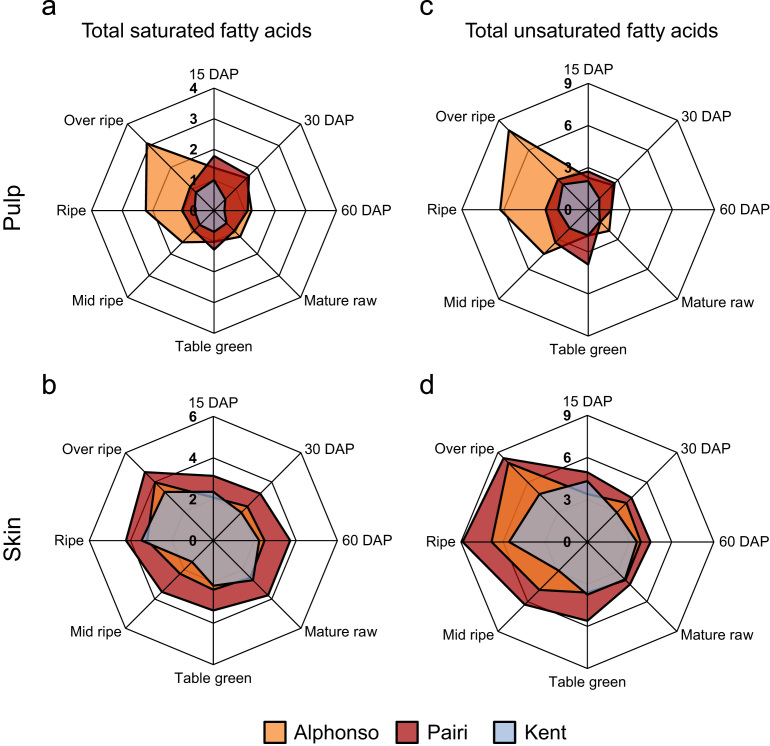
Radar plot representing contribution (mg g^−1^) of total fatty acids. Total saturated fatty acids from pulp (a), total saturated fatty acids from skin (b), total unsaturated fatty acids from pulp (c) and total unsaturated fatty acids from skin (d) at various developing and ripening stages of Alphonso, Pairi and Kent mango cultivars.

A total of 17 different fatty acids were identified and quantified from the pulp and the skin (Table 1, Table 2 and Fig. 1, Fig. 2) at various stages of mango fruit development and ripening from three cultivars *viz*. Alphonso, Pairi and Kent with high, low and no lactone content at ripe stage, respectively (Table 3). Present analysis revealed the ratio of ω6/ω3≤1 at ripe stage (Table 4) suggesting ripened mango fruits as perfect source of essential fatty acids [1].

In the present data, a decrease in C16:0/C16:1 ratio and increase in the fatty acid derived flavor compounds, lactones, were evinced [2], [3] from Alphonso pulp and skin and Pairi pulp (Table 4). Similarly, palmitoleic acid, 11-octadecenoic acid and 9, 15- octadecadienoic acid showed strong correlations with total lactone content from ripe pulp and skin of three cultivars (Table 5), whereas various unsaturated fatty acids showed strong correlation with content of all the eight lactones individually (Table 6).

## 2 Experimental design, materials and methods

### 2.1 Plant material

Fruits of cv. Alphonso and cv. Pairi were collected from the Mango Research Sub Centre, Deogad (16° 31′ N, 73° 20′ E) and of cv. Kent from the Regional Fruit Research Station, Vengurle (15° 51′ N, 73° 39′ E), both affiliated to Dr. Balasaheb Sawant Konkan Agricultural University, Dapoli, Maharashtra, India. Developing stages of all the three mango cultivars were collected at 15, 30 and 60 days after pollination (DAP) and at mature raw stage (90 DAP for cvs. Alphonso and Pairi, 110 DAP for cv. Kent). Fruits at these developing stages were harvested, pulp (mesocarp) and skin (exocarp) separated immediately, snap frozen in liquid nitrogen and stored at −80 °C until further use. A set of 12 fruits each for all the three cultivars were additionally harvested at their respective mature raw stage and kept in the hay containing boxes at ambient temperature for ripening. Three cultivars showed variation in the ripening duration, hence four ripening stages as table green, mid ripe, ripe and over ripe based on the skin color, aroma and fruit softness (each stage is represented by days after harvest i.e. DAH for cv. Alphonso as 5, 10, 15 and 20 days; for cv. Pairi as 4, 6, 8 and 10 days and for cv. Kent as 5, 8, 10 and 13 days, respectively) were used for further analysis. At each ripening stage fruits for each cultivar were removed from the box, pulp and skin were separated, frozen in liquid nitrogen and stored at −80 °C till further use.

### 2.2 Transesterification of fatty acids

Fatty acid methyl esters (FAMEs) were synthesized by transesterification reaction in methanolic HCl. 500 mg of the tissue was finely crushed in liquid nitrogen and added to the 5 ml methanol containing 3 M HCl, 25 µg butylated hydroxyltoluene (BHT) as an antioxidant and 250 µg tridecanoic acid as an internal standard. Transesterification was carried out at 80 °C in water bath for 2 h to synthesize FAMEs. After incubation reaction mixture was cooled on ice and FAMEs were extracted twice in 2 ml n-Hexane. n-Hexane layer was completely evaporated in vacuum evaporator, FAMEs were reconstituted in 250 µl chloroform and used for Gas Chromatography-Mass Spectrometry (GC–MS) and GC–Flame Ionization Detector (FID) analysis.

### 2.3 Extraction of aroma volatiles

Aroma volatiles were extracted from 2 g pulp and skin of completely ripe fruits of all the 3 cultivars by solvent extraction method using dichloromethane with appropriate concentration of nonyl acetate as an internal standard. Procedures for dehydration of dichloromethane, removal of fats and concentrating extracts were carried out as described earlier [4], [5].

### 2.4 Gas chromatography analysis

#### 2.4.1 Identification and quantification of FAMEs

**Fig. 3:**
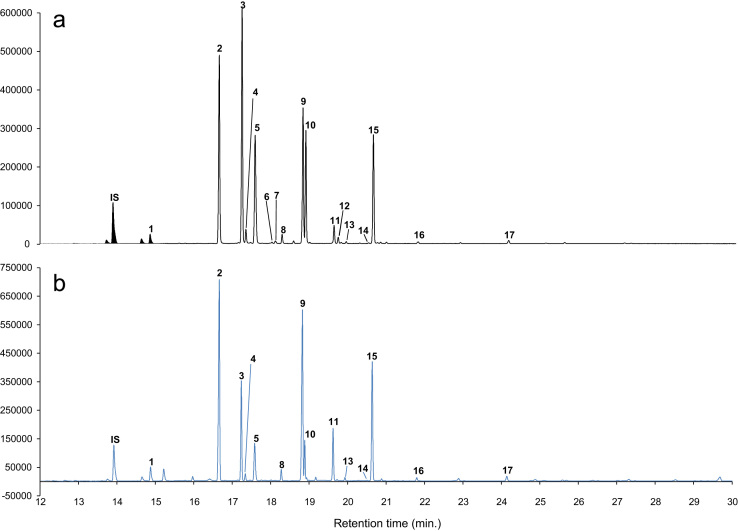
Representative GC chromatogram of fatty acids from pulp (A) and skin (B). Peak labels represents, compounds as IS: Internal standard, 1: Myristic acid, 2: Palmitic acid, 3: Palmitoleic acid, 4: 11-Hexadecenoic acid, 5: Hepta-2,4(E,E)-dienoic acid, 6: 10-Heptadecenoic acid, 7: 9,12-Hexadecadienoic acid, 8: Stearic acid, 9: Oleic acid, 10: 11-Octadecenoic acid, 11: Linoleic acid, 12: 9,15 Octadecanoic acid, 13: Arachidic acid, 14: 11-Eicosenoic acid, 15: α-Linolenic acid, 16: Behenic acid, 17: Lignoceric acid.

**Fig. 4:**
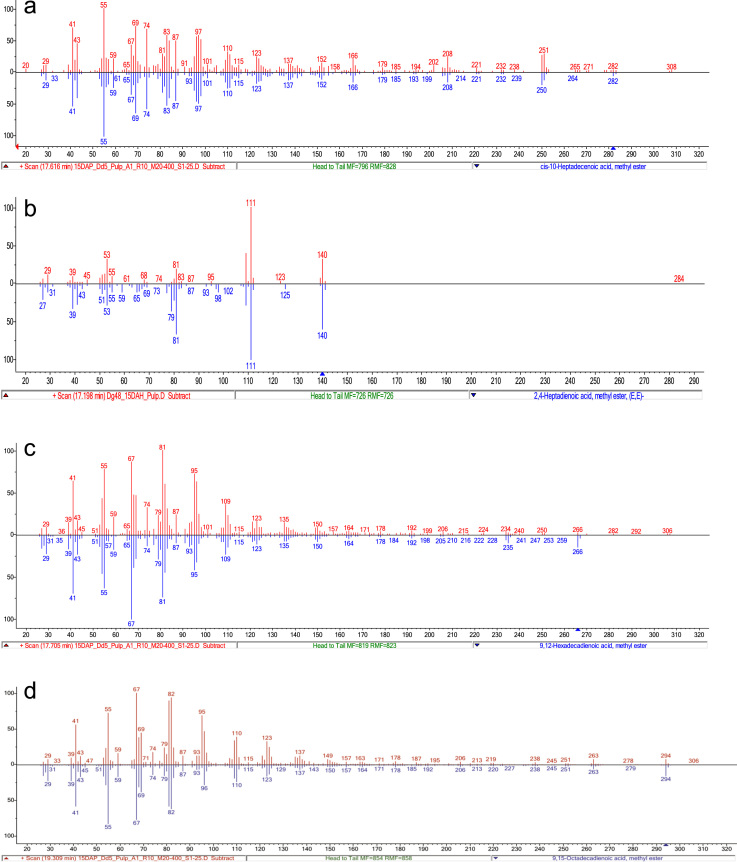
MS spectra of odd chain and unusual poly-unsaturated fatty acid methyl esters. Head to tail alignment of spectra for 10-Heptadecenoic acid (A), Hepta-2,4(E,E)-dienoic acid (B), 9,12 Hexadecadienoic acid (C) and 9,15 Octadecadienoic acid (D). Upward spectra in red color represent experimental spectra while downward spectra in blue color represent standard spectra from NIST 2011 library.

Gas chromatographic analysis was carried out on 7890B GC system Agilent Technologies coupled with Agilent 5977 A MSD (Agilent technologies®, CA, U.S.A.). 1 µl chloroform reconstituted FAMEs were injected for GC–MSD analysis. Method for the gas chromatographic separation of fatty acid structural isoforms was standardized, for better resolution of fatty acids 75 m long SP^™^ 2560 (Supelco, Bellefonte, Pennsylvania, U.S.A.) column with 0.18 mm i.d. and 0.14 µm film thickness was used (Fig. 3). Helium was used as the carrier gas with 1 ml min^−1^ flow. Initial oven temperature was kept at 130 °C and held for 5 min, followed by a ramp of 10 °C min^−1^ till 230 °C with hold at 230 °C for 20 min. Injector temperature was maintained at 250 °C, source, quadrupole and transfer line temperatures were 150 °C, 180 °C and 250 °C, respectively. Mass spectra were obtained by Agilent MSD at 70 eV on scan mode with scanning time of 0.2 s for range of m/z 30–400. FAMEs were identified by matching generated spectra with NIST 2011 and Wiley 10th edition mass spectral libraries (Fig. 4). Identified compounds were confirmed by matching retention time and spectra of authentic standards procured from Sigma Aldrich (St. Louis, MO, USA). Identified compounds were quantified by GC–FID. Similar chromatographic conditions were maintained for GC–FID with detector temperature at 250 °C. Absolute quantification was done using internal standard by normalizing concentrations of all the FAMEs with that of tridecanoic acid methyl ester.

#### 2.4.2 Qualitative and quantitative analysis of lactones

GC-MSD and GC-FID analysis for lactones was carried out on similar instrument used for analysis of FAMEs. Aroma volatiles were separated on GsBP-5MS^®^ (GeneralSeparation Technologies, Newark, DE, U.S.A.) capillary column (30 m×0.32 mm i.d. ×0.25 µm film thickness). Other chromatographic conditions were maintained as mentioned previously [6]. Since fatty acids are known to be the precursors for lactone biosynthesis, qualitative and quantitative analysis for lactones alone was carried out in the present study. Lactones were identified by matching generated spectra with NIST 2011 and Wiley 10^th^ edition mass spectral libraries. Identified compounds were confirmed by matching retention time and spectra of authentic standards procured from Sigma Aldrich (St. Louis, MO, U.S.A.). Absolute quantification was done using internal standard by normalizing concentrations of all the lactones with that of known concentration of nonyl acetate.

### 2.5 Statistical analysis

**Fig. 5:**
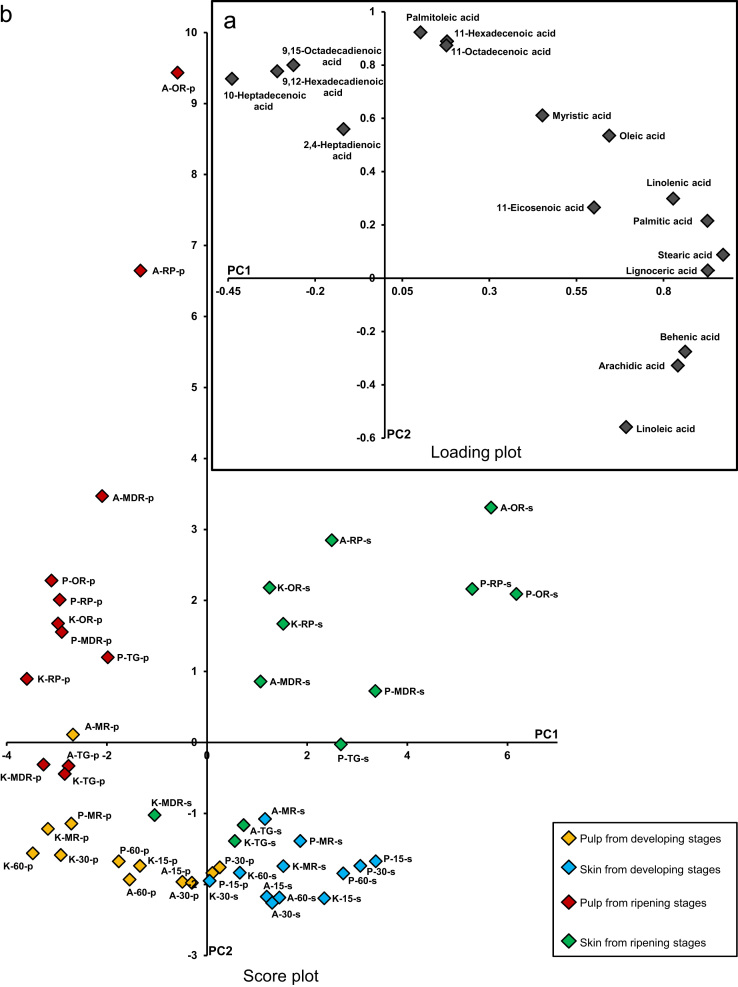
Loading plot (a) and Score plot (b) of principle component analysis of 17 different fatty acids contributing to pulp and skin through various stages of fruit development and ripening from three mango cultivars, Alphonso, Pairi and Kent. Average values from biological replicates for each fatty acid were considered for the analysis. For the score plot (b) data labels represents, cultivars as A: Alphonso, P: Pairi and K: Kent, tissues as p: pulp and s: skin and stages as15: 15DAP, 30: 30DAP, 60: 60DAP, MR: mature raw, TG: table green, MDR: mid ripe, R: ripe and OR: over ripe.

To validate data statistically tissue for each developing and ripening stages were collected from fruits of 3 independent trees for cv. Alphonso and 2 independent trees each for cv. Pairi and cv. Kent. These were considered as biological replicates. Extraction of FAMEs and volatiles was carried out twice for each tissue as technical replicates followed by duplicate GC–FID runs of each extracts as analytical replicates. Fisher׳s LSD test was performed separately for pulp and skin at *p*≤0.05 by ANOVA for comparative analysis of quantity of each fatty acid during various developing and ripening stages from each cultivar. Also comparison was done for each fatty acid at individual stage among the three cultivars using StatView® software, version 5.0 (SAS Institute Inc., Cary, NC, U.S.A.). Similarly ANOVA was carried out for lactone content of ripe pulp and skin from the three cultivars. Correlation analysis of total lactone content with individual fatty acid content and individual lactone content with individual fatty acid content from the pulp and the skin of three cultivars at ripe stage was studied using StatView software. Principle component analysis for whole data set (Fig. 5) of fatty acid content was carried out using Systat® statistical software (Version11, Richmond, CA, U.S.A.).

## Figures and Tables

**Table 1:** Fatty acid composition of pulp. Fatty acid composition (µg g^−1^ tissue) of pulp at various stages of fruit development and ripening from Alphonso, Pairi and Kent cultivars. Values shown are average of biological replicates sampled for the study. Difference between the stages was significant (*p*≤0.05) if the alphabets (a, b, c….) after the quantity of the compounds are different. Difference between the cultivars for each compound at each stage was significant (*p*≤0.05) if the alphabets (x, y, z) after the quantity of the compounds are different.

**Compound**		**15 DAP**	**30 DAP**	**60 DAP**	**Mature raw**	**Table green**	**Mid ripe**	**Ripe**	**Over ripe**
**Saturated fatty acids**									
Myristic acid^a^ (C14:0)	Alphonso	8.41^a,y^	5.95^a,xy^	6.12^a^	28.81^a,y^	20.68^a,x^	83.09^b,z^	174.29^c,y^	184.41^c,y^
	Pairi	7.2^a,xy^	7.73^a,y^	6.23^a^	24.66^b,xy^	77.54^d,y^	60.29^c,y^	74.03^cd,x^	77.56^d,x^
	Kent	4.24^a,x^	3.8^a,x^	6^a^	5.42^a,x^	16.41^b,x^	26.65^c,x^	40.57^d,x^	52.44^e,x^
Palmitic acid^a^ (C16:0)	Alphonso	1186.80^a,y^	1302.72^a,y^	1073.10^a,y^	1097.93^a,z^	922.36^a,y^	1274.21^a,y^	1933.43^b,y^	2705.91^c,y^
	Pairi	1534.97^b,z^	1347.69^b,y^	962.06^a,y^	843.77^a,y^	1117.85^ab,y^	791.2^a,x^	896^a,x^	946.51^a,x^
	Kent	830.61^e,x^	456.04^b,x^	290.26^a,x^	517.62^bc,x^	597.42^c,x^	574.87^c,x^	560.88^c,x^	678^d,x^
Stearic acid^a^ (C18:0)	Alphonso	67.97^b,y^	62.12^b,y^	54.25^ab,*y*^	41.49^ab,z^	36.07^a^	50.28^ab,y^	75.63^b,y^	115.93^c,y^
	Pairi	84.9^b,z^	81.52^b,y^	49.14^a,y^	26.92^a,y^	39.04^a^	30.13^a,xy^	33.36^a,x^	36.74^a,x^
	Kent	57.29^d,x^	21.92^ab,x^	19.29^a,x^	20.18^ab,x^	40.05^c^	23.05^ab,x^	29.76^b,x^	25.27^ab,x^
Arachidic acid^a^ (C20:0)	Alphonso	32.61^c,y^	33.66^c,y^	23.93^bc,y^	10.75^a^	9.97^a^	10.65^a,y^	19.01^b,z^	27.07^c,y^
	Pairi	43.61^c,z^	47.16^c,y^	24.24^b,y^	11.42^ab^	8.53^a^	7.52^a,xy^	7.2^a,y^	6.25^a,x^
	Kent	19.89^c,x^	7.91^b,x^	4.78^ab,x^	8.4^b^	7.38^b^	5.78^ab,x^	3.2^a,x^	6.47^b,x^
Behenic acid^a^ (C22:0)	Alphonso	52.78^c,y^	49.96^c,y^	33.10^b^	8.06^a^	8.22^a^	14.33^ab,y^	24.90^b,y^	33.21^b,y^
	Pairi	62.12^c,y^	61.13^c,y^	24.70^b^	9.30^a^	7.36^a^	8.34^a,xy^	8.88^a,x^	8.33^a,x^
	Kent	37.82^c,x^	13.97^b,x^	6.50^ab^	8.66^ab^	8.02^ab^	5.74^ab,x^	3.67^a,x^	9.02^b,x^
Lignoceric acid (C24:0)	Alphonso	53.35^c^	50.46^c,xy^	33.89^b,y^	18.00^a^	20.06^a^	27.97^ab^	35.85^bc.y^	41.46^bc^
	Pairi	52.36^b^	67.32^b,y^	33.18^ab,xy^	18.30^a^	23.23^a^	23.60^a^	27.04^a,x^	28.39^a^
	Kent	48.53^ab^	16.89^a,x^	9.90^a,x^	17.25^a^	24.87^a^	23.86^a^	24.88^a.x^	69.49^b^

**Mono-unsaturated fatty acids**									
Palmitoleic acid^a^ (C16:1, n-7)	Alphonso	11.79^a,x^	9.15^a,x^	11.79^a,x^	463.26^a,y^	293.57^a,x^	1325.77^b,y^	2881.90^c,y^	4325.90^d,y^
	Pairi	15.07^a,y^	15.77^a,xy^	21.6^a,xy^	188.54^a,x^	715.89^b,y^	530.07^b,x^	599.84^b,x^	586.91^b,x^
	Kent	13.52^a,xy^	18.11^a,y^	29.38^a,y^	77.29^b,x^	147.53^c,x^	201.97^d,x^	314.28^e,x^	452.73^f,x^
11-Hexadecenoic acid^b^ (C16:1, n-5)	Alphonso	n.d.	n.d.	n.d.	20.84^b^	12.83^ab,x^	74.79^c,y^	146.22^d,z^	174.44^e,y^
	Pairi	n.d.^a^	n.d.^a^	n.d.^a^	14.71^b^	59.37^c,y^	45.13^c,xy^	51.49^c,y^	45.41^c,x^
	Kent	n.d.^a^	n.d.^a^	n.d.^a^	2.15^b^	8.95^c,x^	14.87^d,x^	22.42^e,x^	31.81^f,x^
10-Heptadecenoic acid^a^, ^b^ (C17:1, n-7)	Alphonso	n.d.^a^	n.d.^a^	n.d.^a^	6.93^b,z^	6.10^b,y^	7.84^bc^	11.82^c,z^	18.69^d,y^
	Pairi	n.d.^a^	n.d.^a^	n.d.^a^	n.d.^a,x^	n.d.^a,x^	5.15^b^	8.76^c,y^	7.94^c,x^
	Kent	n.d.^a^	n.d.^a^	n.d.^a^	2.08^b,y^	4.31^c,y^	4.31^c^	3.76^c,x^	5.64^d,x^
Oleic acid^a^ (C18:1, n-9)	Alphonso	271.54^ab,xy^	222.82^ab,xy^	161.31^a,x^	412.00^b,y^	357.16^ab,x^	759.02^c,y^	856.59^c,y^	1117.90^d,y^
	Pairi	328.43^a,y^	463.79^ab,y^	282.07^a,y^	364.83^a,y^	1119.48^c,y^	765.78^b,y^	761.79^b,y^	683.93^b,xy^
	Kent	255.24b^c,x^	133.62^ab,x^	97.01^a,x^	198.28^b,x^	285.4^c,x^	304.1^c,x^	261.3^bc,x^	296.77^c,x^
11-Octadecenoic acid^a^, ^b^ (C18:1, n-7)	Alphonso	29.06^a,x^	32.88^a^	33.01^a,x^	364.47^b,y^	289.59^b^	590.53^c,y^	646.48^c,y^	767.85^c,y^
	Pairi	112.82^ab,z^	103.02^ab^	85.8^a,y^	144.54^ab,x^	427.63^c^	268.46^b,x^	248.78^b,x^	199.9^b,x^
	Kent	46.48^a,y^	45.21^a^	41.69^a,xy^	102.89^b,x^	210.1^cd^	177.94^c,x^	176.61^c,x^	254.79^d,x^
11-Eicosenoic acid^b^ (C20:1, n-9)	Alphonso	6.59^b^	9.04^bc^	4.92^ab^	1.65^a,x^	1.94^a^	3.44^ab,y^	6.57^b,y^	10.24^c,y^
	Pairi	6.9^bc^	8.67^c^	5.09^b^	1.97^a,xy^	3.04^ab^	2.5^a,xy^	2.39^a,x^	2.06^a,xy^
	Kent	5.87^c^	4.67^c^	2.48^b^	2.72^b,y^	3.06^b^	n.d.^a,x^	n.d.^a,x^	n.d.^a,x^

**Poly-unsaturated fatty acid**									
9,12 Hexadecadienoic acid^b^ (C16:2, n-4)	Alphonso	n.d.^a^	n.d.^a^	n.d.^a^	n.d.^a^	n.d.^a^	15.38^b,y^	33.86^c,y^	52.02^d^
	Pairi	n.d.^a^	n.d.^a^	n.d.^a^	n.d.^a^	n.d.^a^	9.12^b,xy^	17.71^b,x^	26.7^d^
	Kent	n.d.^a^	n.d.^a^	n.d.^a^	n.d.^a^	n.d.^a^	n.d.^a,x^	16.08^b,x^	22.91^c^
Linoleic acid^a^ (C18:2, n-6)	Alphonso	1425.06^cd,xy^	1707.67^d,y^	1119.71^c^	415.66^ab^	471.57^b,y^	115.73^a,x^	83.58^a,x^	79.46^a^
	Pairi	1699.08^c,y^	1575.41^c,y^	953.05^b^	400.89^a^	198.49^a,x^	138.39^a,xy^	139.44^a,y^	95.55^a^
	Kent	1178.94^e,x^	546.53^d,x^	260.25^b^	388.18^c^	387.2^c,y^	158.96^a,y^	80.05^a,x^	95.98^a^
9,15 Octadecadienoic acid^b^ (C18:2, n-3)	Alphonso	n.d.^a^	n.d.^a^	n.d.^a^	n.d.^a^	n.d.^a^	30.20^b,y^	61.58^c,y^	85.94^d,y^
	Pairi	n.d.^a^	n.d.^a^	n.d.^a^	n.d.^a^	n.d.^a^	12.95^b,xy^	20.24^bc,x^	24.3^c,x^
	Kent	n.d.^a^	n.d.^a^	n.d.^a^	n.d.^a^	n.d.^a^	n.d.^a,x^	20.93^b,x^	28.09^c,x^
Hepta-2,4(E,E)-dienoic acid^b^ (C7:2, n-3)	Alphonso	79.55^a,x^	54.86^a,x^	52.45^a,x^	117.22^a,x^	80.16^a,x^	727.71^c^	698.01^c^	481.16^b^
	Pairi	167.66^a,y^	196.83^a,y^	120.89^a,y^	120.23^a,x^	954.79^b,z^	1120.84^b^	662.32^ab^	841.11^b^
	Kent	215.1^a,z^	233.39^a,y^	248.32^a,z^	247.62^a,y^	441.4^b,y^	604.7^c^	835.33^e^	707.57^d^
Linolenic acid^a^ (C18:3, n-3)	Alphonso	454.06^b,y^	443.29^b,y^	270.27^a^	323.19^a,z^	327.86^ab^	803.64^c,y^	840.37^c,y^	881.25^c,y^
	Pairi	394.56^c,xy^	301.43^b,xy^	202.99^a^	237.76^ab.y^	424.59^c^	410.51^c,x^	522.23^d,x^	532.25^d,x^
	Kent	326.21^b,x^	142.55^a,x^	106.54^a^	158.43^a,x^	343.86^b^	402.79^b,x^	408.42^b,x^	653.13^c,x^

n.d.: not detected.

*a* Compounds identified by matching mass spectrum from NIST2011 and Wiley 10th edition mass spectral libraries and retention time and mass spectrum of authentic standard; remaining compounds were identified by matching mass spectrum from NIST2011 and Wiley 10th edition mass spectral libraries. MS spectra for odd chain and unusual poly-unsaturated FAMEs has been provided as Fig. 4.

*b* Unusual fatty acids.

**Table 2:** Fatty acid composition of skin. Fatty acid composition (µg g^−1^ tissue) of skin at various stages of fruit development and ripening from Alphonso, Pairi and Kent cultivars. Values shown are average of biological replicates sampled for the study. Difference between the stages was significant (*p*≤0.05) if the alphabets (a, b, c….) after the quantity of the compounds are different. Difference between the cultivars for each compound at each stage was significant (*p*≤0.05) if the alphabets (x, y, z) after the quantity of the compounds are different.

**Compound**		**15 DAP**	**30 DAP**	**60 DAP**	**Mature raw**	**Table green**	**Mid ripe**	**Ripe**	**Over ripe**
**Saturated fatty acids**									
Myristic acid^a^ (C14:0)	Alphonso	17.47^a,xy^	14.84^a,y^	14.6^a,xy^	49.79^a,y^	42.67^a,y^	145.71^b,y^	231.21^c,x^	216.96^c,x^
	Pairi	19.58^a,y^	19.56^a.y^	17.49^a,y^	23.95^a,x^	92.42^ab,z^	168.01^b,y^	295.16^c,xy^	387.91^d,y^
	Kent	9.96^a,x^	8.99^a,x^	11.58^a,x^	12.58^a,x^	22.49^b,x^	51.14^c,x^	323.9^d,y^	538.8^e,z^
Palmitic acid^a^ (C16:0)	Alphonso	1738.73^a,x^	2011.49^a,y^	2139.59^ab,x^	2226.01^ab,x^	2088.43^a,x^	1890.39^a,y^	2682.16^b^	3253.66^c,y^
	Pairi	2653.33^a,y^	2768.19^ab,z^	3309.13^b,y^	3407.48^b,y^	2938.97^ab,y^	2958.08^ab,z^	3460.13^b^	3756.19^b,y^
	Kent	1944.53^c,x^	1637.99^b,x^	1921.57^c,x^	2338.6^d,x^	1898^c,x^	1215.83^a,x^	2883.29^f^	2534.02^e,x^
Stearic acid^a^ (C18:0)	Alphonso	120.07^a,x^	108.78^a,x^	122.28^a,xy^	104.3^a,x^	103.52^a,x^	92.13^a,x^	123.57^a,x^	189.51^b,y^
	Pairi	172.83^a,y^	168.75^a,y^	153.24^a,y^	135.52^a,y^	156.64^a,y^	166.39^a,y^	238.57^b,y^	263.01^b,z^
	Kent	130.25^c,x^	93.98^ab,x^	103.75^b,x^	150.81^d,y^	90.88^ab,x^	82.32^a,x^	116.39^bc,x^	116.26^bc,x^
Arachidic acid^a^ (C20:0)	Alphonso	58.52^b^	53.18^b,x^	58.98^b^	54.67^b,x^	48.66^b^	31.08^a,x^	29.21^a,x^	48.28^b,xy^
	Pairi	80.59^b^	86.02^b,y^	81.04^b^	64.21^ab,xy^	55.21^a^	56.9^a,y^	55.24^a,y^	60.27^a,y^
	Kent	61.9^c^	50.78^b,x^	59.04^bc^	72.13^d,y^	58.39^bc^	28.13^a,x^	32.56^a,x^	26.99^a,x^
Behenic acid^a^ (C22:0)	Alphonso	66.96^bc,x^	65.62^b,y^	66.31^b,xy^	45.24^a,x^	41.88^a^	40.38^a,y^	43.83^a^	83.07^c,y^
	Pairi	90.76^b,xy^	86.38^b,z^	76.46^b,y^	56.30^a,xy^	53.27^a^	54.01^a,z^	55.38^a^	60.04^ab,xy^
	Kent	94.21^d,y^	50.79^bc,x^	52.60^bc,x^	59.86^c,y^	49.52^bc^	29.01^a,x^	43.73^b^	46.97^b,x^
Lignoceric acid (C24:0)	Alphonso	80.13^a,x^	76.08^a^	76.42^a^	59.20^a,x^	53.98^a,x^	72.76^a,xy^	86.16^a^	192.83^b,y^
	Pairi	110.84^b,y^	101.31^ab^	82.83^ab^	60.52^a,xy^	85.25^ab,y^	110.56^b,y^	117.24^b^	149.49^b,y^
	Kent	115.19^c,y^	68.11^b^	72.40^b^	79.44^b,y^	66.49^b,x^	50.17^a,x^	71.15^b^	62.16^ab,x^

**Mono-unsaturated fatty acids**									
Palmitoleic acid^a^ (C16:1, n-7)	Alphonso	32.95^a,x^	24.95^a,x^	34.95^a,x^	150.86^a^	152.43^a,x^	736.63^b,y^	1986.59^c,y^	2266.38^c,z^
	Pairi	50.48^a,y^	46.57^a,y^	55.57^a,y^	75.68^a^	189.08^b,y^	296.74^c,x^	533.59^d,x^	697.64^e,x^
	Kent	48.93^a,y^	60.04^a,z^	91.5^ab,z^	135.47^b^	155.83^b,x^	170.38^b,x^	1527.72^c,y^	1621.55^d,y^
11-Hexadecenoic acid^b^ (C16:1, n-5)	Alphonso	n.d.^a^	n.d.^a^	n.d.^a^	7.66^b,y^	7.1^b,x^	45.8^c,y^	119.07^d,y^	120.18^d,y^
	Pairi	n.d.^a^	n.d.^a^	n.d.^a^	8.4^b,y^	19.06^c,y^	33.24^d,xy^	58.01^e,x^	78.76^f,x^
	Kent	n.d.^a^	n.d.^a^	n.d.^a^	n.d.^a,x^	10.84^b,x^	14.11^b,x^	147.05^c,y^	160.5^d,z^
10-Heptadecenoic acid^a^^,^^b^ (C17:1, n-7)	Alphonso	n.d.	n.d.	n.d.	n.d.	n.d.	n.d.	n.d.	n.d.
	Pairi	n.d.	n.d.	n.d.	n.d.	n.d.	n.d.	n.d.	n.d.
	Kent	n.d.	n.d.	n.d.	n.d.	n.d.	n.d.	n.d.	n.d.
Oleic acid^a^ (C18:1, n-9)	Alphonso	299.97^a,x^	251.48^a,x^	278.99^a,x^	785.17^ab,y^	622.61^a,x^	1532.67^b,y^	2376.3^c,y^	2982.71^c,y^
	Pairi	394.18^a,y^	451.32^a,y^	426.62^a,z^	719.85^b,xy^	1604.09^c,y^	1764.73^c,y^	2847.25^d,y^	2587.84^d,y^
	Kent	343.36^b,xy^	241.6^a,x^	349.46^b,y^	500.19^c,x^	461.87^c,x^	310.41^b,x^	778.48^e,x^	700.8^d,x^
11-Octadecenoic acid^a^^,^^b^ (C18:1, n-7)	Alphonso	43.86^a,x^	54.94^a,x^	71.09^a,x^	179.86^ab,xy^	181.97^ab,x^	298.65^b,y^	480.59^c,y^	589.91^c,y^
	Pairi	341.82^c,z^	298.55^bc,z^	225.38^a,z^	213.48^a,y^	231.02^a,y^	253.48^ab,y^	321.16^c,x^	305.35^bc,xy^
	Kent	103.73^b,y^	71.75^a,y^	94.98^ab,y^	133.16^bc,x^	142.92^c,x^	93.29^ab,x^	282.14^e,x^	243.65^d,x^
11-Eicosenoic acid^b^ (C20:1, n-9)	Alphonso	3.71^a,x^	4.79^a,y^	3.89^a,y^	5.16^a,y^	4.03^a,y^	6.98^ab,y^	10.01^b,y^	15.24^c,y^
	Pairi	6.65^b,y^	5.92^ab,z^	4.33^ab,y^	3.26^a,x^	5.88^ab,z^	8.37^bc,y^	10.49^b,y^	8.92^bc,y^
	Kent	4.78^e,x^	2^d,x^	1.66^cd,x^	2.23^d,x^	1.31^c,x^	0.49^b,x^	n.d.^a,x^	n.d.^a,x^

**Poly-unsaturated fatty acid**									
9,12 Hexadecadienoic acid^b^ (C16:2, n-4)	Alphonso	n.d.	n.d.	n.d.	n.d.	n.d.	n.d.	n.d.	n.d.
	Pairi	n.d.	n.d.	n.d.	n.d.	n.d.	n.d.	n.d.	n.d.
	Kent	n.d.	n.d.	n.d.	n.d.	n.d.	n.d.	n.d.	n.d.
Linoleic acid^a^ (C18:2, n-6)	Alphonso	2054.46^c^	2671.6^d,y^	2446.85^cd,xy^	1385.42^b,x^	1374.07^b^	476.06^a,x^	422.83^a,x^	535.69^a,x^
	Pairi	3104.44^c^	2742.04^bc,y^	2808.19^bc,y^	2223.28^b,y^	1516.53^a^	1552.89^a,z^	1956.03^ab,z^	1941.42^ab,z^
	Kent	2749.59^e^	1735.03^cd,x^	1860.57^d,x^	1953.45^d,y^	1583.19^c^	918.49^a,y^	1277.41^b,y^	856.52^a,y^
9,15 Octadecadienoic acid^b^ (C18:2, n-3)	Alphonso	n.d.	n.d.	n.d.	n.d.	n.d.	n.d.	n.d.	n.d.
	Pairi	n.d.	n.d.	n.d.	n.d.	n.d.	n.d.	n.d.	n.d.
	Kent	n.d.	n.d.	n.d.	n.d.	n.d.	n.d.	n.d.	n.d.
Hepta-2,4(E,E)-dienoic acid^b^ (C7:2, n-3)	Alphonso	29.95^a,x^	28.07^a,x^	31.6^a,x^	63.06^a,x^	60.94^a,x^	426^d,x^	265.93^c,x^	142.11^b,x^
	Pairi	190.16^a,z^	170.78^a,y^	166.88^a,y^	166.65^a,y^	563.59^b,z^	901.98^c,z^	1152.72^d,y^	621.23^b,z^
	Kent	158.38^a,y^	258.62^b,z^	261.23^b,z^	271.33^b,z^	346.22^c,y^	553.56^d,y^	352.98^c,x^	335.56^c,y^
Linolenic acid^a^ (C18:3, n-3)	Alphonso	938.51^ab^	883.07^a,z^	931.97^ab^	1265.3^b,y^	1196.94^ab,xy^	1303.57^b,y^	1149.88^ab,x^	1299.23^b,x^
	Pairi	866.49^a^	724.08^a,y^	805.88^a^	868.83^a,x^	1480.56^b,y^	1487.2^b,y^	1991.68^c,y^	2181.91^c,y^
	Kent	918.68^c^	590.34^a,x^	869.18^bc^	778.13^b,x^	1048.87^d,x^	821.21^bc,x^	1201.18^e,x^	897.43^bc,x^

n.d.: not detected.

*a* Compounds identified by matching mass spectrum from NIST2011 and Wiley 10th edition mass spectral libraries and retention time and mass spectrum of authentic standard; remaining compounds were identified by matching mass spectrum from NIST2011 and Wiley 10th edition mass spectral libraries.

*b* Unusual fatty acids.

**Table 3:** Lactone content of ripe fruit. Lactone content (µg g^−1^ tissue) of pulp and skin of Alphonso, Pairi and Kent at ripe stage.

Lactone	Alphonso	Alphonso	Pairi	Pairi	Kent	Kent
	pulp	skin	pulp	skin	pulp	skin
γ-Butyrolactone	1.39±0.16	0.17±0.01	0.30±0.03	0.09±0.01	Trace^a^	Trace^a^
γ-Hexalactone	1.45±0.16	0.28±0.01	0.12±0.04	n.d.	n.d.	n.d.
δ-Hexalactone	1.07±0.17	0.13±0.02	0.71±0.05	0.26±0.01	n.d.	n.d.
γ-Octalactone	2.16±0.20	1.49±0.09	0.07±0.01	0.28±0.03	n.d.	n.d.
δ-Octalactone	0.65±0.05	0.15±0.002	0.11±0.03	0.14±0.01	n.d.	n.d.
γ-Decalactone	0.32±0.28	0.33±0.04	n.d.	0.35±0.06	n.d.	n.d.
δ-Decalactone	0.09±0.01	0.61±0.20	n.d.	n.d.	n.d.	n.d.
Total	7.12	3.16	1.3	1.12	n.d.	n.d.

a Compound detected in traces in GC–MS analysis but not detected in GC–FID analysis.

**Table 4:** Flavor and nutritional perspective of fatty acids. Table representing total lactone content (µg g^−1^), palmitic acid/palmitoleic acid (16:0/C16:1) ratio and linoleic acid/linolenic acid (ω6/ω3) in pulp and skin at ripe stage of Alphonso, Pairi and Kent mango cultivars. Values shown are average of biological replicates sampled for the study. Difference between the tissues was significant (*p*≤0.05) if the alphabets (a, b, c….) after the quantity of the lactone are different.

	Flavor		Nutrition
Ripe tissue	Total lactone	C16:0/C16:1	LA/ALA (ω6/ω3)
	content		
Alphonso pulp	7.12^d^	0.67	0.1
Pairi pulp	1.30^b^	1.49	0.27
Kent pulp	nd^a^	1.78	0.2
Alphonso skin	3.16^c^	1.35	0.37
Pairi skin	1.12^b^	6.48	0.98
Kent skin	nd^a^	1.89	1.06

**Table 5:** Correlation analysis. Correlation analysis of total lactone content (µg g^−1^ tissue) and individual fatty acid content from the pulp and the skin tissues of Alphonso, Pairi and Kent cultivars at ripe stage. Values represent correlation coefficient (*r*), Values in bold represents strong positive correlation (0.7≤*r*) between fatty acid and lactone.

Fatty acid	Correlation coefficient
Myristic acid	−0.008
Palmitic acid	0.064
Stearic acid	−0.064
Arachidic acid	−0.043
Behenic acid	0.027
Lignoceric acid	−0.144
Palmitoleic acid	**0.847**
11-Hexadecenoic acid	0.547
10-Heptadecenoic acid	0.633
Oleic acid	0.078
11-Octadecenoic acid	**0.954**
11-Eicosenoic acid	0.477
9,12-Hexadecadienoic acid	0.645
Linoleic acid	−0.385
9,15-Octadecadienoic acid	**0.76**
2,4-Heptadienoic acid	−0.099
Linolenic acid	−0.053

**Table 6:** Correlation analysis. Correlation analysis of individual lactone and individual fatty acid content from the pulp and the skin tissues of Alphonso, Pairi and Kent cultivars at ripe stage. Values represent correlation coefficient (*r*), Values in bold represents strong positive correlation (0.7≤*r*) between fatty acid and lactone content.

Lactone	Palmitoleic acid	10-Heptadecenoic acid	11-Octadecenoic acid	11-Eicosenoic acid	9,12-Hexadecadienoic acid	9,15-Octadecadienoic acid
γ-Butyrolactone	**0.752**	**0.813**	**0.828**	0.229	**0.823**	**0.91**
γ-Hexalactone	**0.832**	**0.73**	**0.885**	0.259	**0.77**	**0.878**
δ-Hexalactone	0.487	**0.9**	0.622	0.202	**0.808**	**0.825**
γ-Octalactone	**0.888**	0.416	**0.974**	0.566	0.457	0.592
δ-Octalactone	**0.77**	**0.706**	**0.893**	0.405	**0.724**	**0.836**
γ-Decalactone	0.496	−0.058	**0.748**	**0.953**	−0.035	0.118
δ-Decalactone	0.452	−0.283	0.472	0.558	−0.287	−0.22
